# Supplementary material for: Salivary Metabolomic Analysis Reveals Amino Acid Metabolism Shift in SARS-CoV-2 Virus Activity and Post-Infection Condition
Source: Metabolites. 2023 Feb 11;13(2):263. doi: 10.3390/metabo13020263 (PMC9962089; doi:10.3390/metabo13020263)
Supplement: Supplementary file 1 [file metabolites-13-00263-s001.zip › metabolites-2172553-supplementary.pdf]

SUPPLEMENTAL MATERIAL

Supplementary Table S1: Saliva metabolite intensities shown as the mean (arbitrary units), confidence interval, bucket region (ppm), and statistical analysis from multivariate analysis using the [I]loading factor and [II]univariate analysis.

Metabolites	Chemical shift	PCR-[a] Mean (95%CI)	PCR+[b] Mean (95%CI)	Post Sars-CoV-2 infection[c] Mean (95%CI)	*p*-value
Acetate[I,II] HMDB00042	1.92	180.4 x 10^{-3} (27.7 x 10^{-3} – 375.7 x 10^{-3})	29.1 x 10^{-3} (1.0 x 10^{-3} – 71.9 x 10^{-3})	8.8 x 10^{-3} (2.4 x 10^{-3} – 26.3 x 10^{-3})	<0.001[ab] <0.001[ac]
Alanine[I] HMDB00161	1.46	3.9 x 10^{-3} (1.2 x 10^{-3} – 7.1 x 10^{-3})	3.6 x 10^{-3} (0.5 x 10^{-3} – 4.9 x 10^{-3})	2.7 x 10^{-3} (0.8 x 10^{-3} – 7.8 x 10^{-3})	ND
Aminobutyrate[I] HMDB00112	1.83	3.1 x 10^{-3} (0.9 x 10^{-3} – 5.4 x 10^{-3})	2.3 x 10^{-3} (0.1 x 10^{-3} – 4.2 x 10^{-3})	1.8 x 10^{-3} (0.0 x 10^{-3} – 3.3 x 10^{-3})	ND
Butyrate[I] HMDB00039	1.11	0.9 x 10^{-3} (0.4 x 10^{-3} – 37.1 x 10^{-3})	0.5 x 10^{-3} (0.0 x 10^{-3} – 69.0 x 10^{-3})	1.0 x 10^{-3} (0.0 x 10^{-3} – 20.1 x 10^{-3})	ND
Caproic acid[I,II] HMDB00535	0.84	3.0 x 10^{-3} (0.0 x 10^{-3} – 5.5 x 10^{-3})	0.6 x 10^{-3} (0.0 x 10^{-3} – 1.3 x 10^{-3})	1.3 x 10^{-3} (0.2 x 10^{-3} – 3.5 x 10^{-3})	0.003[ab] 0.007[bc]
Ethanol[I, II] HMDB00108	1.17	11.8 x 10^{-3} (6.5 x 10^{-3} – 34.2 x 10^{-3})	6.3 x 10^{-3} (4.1 x 10^{-3} – 20.6 x 10^{-3})	12.6 x 10^{-3} (4.1 x 10^{-3} – 73.2 x 10^{-3})	ND
Formic acid[II] HMDB00142	8.4	0.00 x 10^{-3} (0.0 x 10^{-3} – 0.1 x 10^{-3})	0.1 x 10^{-3} (0.0 x 10^{-3} – 0.5 x 10^{-3})	0.1 x 10^{-3} (0.0 x 10^{-3} – 0.3 x 10^{-3})	0.05[ab]
Glucose[I] HMDB0304632	3.84	9.8 x 10^{-3} (3.9 x 10^{-3} – 54.5 x 10^{-3})	4.9 x 10^{-3} (2.7 x 10^{-3} – 98.8 x 10^{-3})	6.4 x 10^{-3} (3.2 x 10^{-3} – 15.4 x 10^{-3})	ND
Glutamine[I] HMDB00641	3.81	9.7 x 10^{-3} (4.7 x 10^{-3} – 23.0 x 10^{-3})	5.3 x 10^{-3} (3.2 x 10^{-3} – 34.7 x 10^{-3})	6.7 x 10^{-3} (3.4 x 10^{-3} – 13.7 x 10^{-3})	ND
Histidine[II] HMDB0003412	7.03	1.5 x 10^{-3} (0.2 x 10^{-3} – 2.5 x 10^{-3})	0.6 x 10^{-3} (0.3 x 10^{-3} – 1.3 x 10^{-3})	0.9 x 10^{-3} (0.4 x 10^{-3} – 1.5 x 10^{-3})	0.015[ab]
Hydroxylysine[I] HMDB0000450	2.91	1.0 x 10^{-3} (0.6 x 10^{-3} – 6.0 x 10^{-3})	3.4 x 10^{-3} (0.8 x 10^{-3} – 6.5 x 10^{-3})	1.1 x 10^{-3} (0.5 x 10^{-3} – 5.7 x 10^{-3})	ND
Lactate[I,II] HMDB00190	1.32	6.8 x 10^{-3} (1.5 x 10^{-3} – 23.2 x 10^{-3})	4.2 x 10^{-3} (1.6 x 10^{-3} – 54.7 x 10^{-3})	8.7 x 10^{-3} (2.5 x 10^{-3} – 34.4 x 10^{-3})	ND
Leucine[II] HMDB00687	1.76	4.0 x 10^{-3} (2.7 x 10^{-3} – 5.8 x 10^{-3})	3.2 x 10^{-3} (0.1 x 10^{-3} – 4.3 x 10^{-3})	2.0 x 10^{-3} (0.0 x 10^{-3} – 3.3 x 10^{-3})	0.030[ab] 0.007[ac]
Lysine[II] HMDB03405	1.73	4.7 x 10^{-3} (2.0 x 10^{-3} – 6.9 x 10^{-3})	3.4 x 10^{-3} (0.2 x 10^{-3} – 5.2 x 10^{-3})	3.1 x 10^{-3} (0.2 x 10^{-3} – 4.7 x 10^{-3})	0.002[ac]
Phenylalanine[II] HMDB0000159	7.38	2.5 x 10^{-3} (0.5 x 10^{-3} – 4.9 x 10^{-3})	1.0 x 10^{-3} (0.3 x 10^{-3} – 1.7 x 10^{-3})	1.4 x 10^{-3} (0.6 x 10^{-3} – 1.5 x 10^{-3})	0.031[ab]
Proline[I] HMDB0000162	3.39	4.3 x 10^{-3} (0.0 x 10^{-3} – 6.6 x 10^{-3})	2.4 x 10^{-3} (0.6 x 10^{-3} – 20.5 x 10^{-3})	2.1 x 10^{-3} (0.7 x 10^{-3} – 10.0 x 10^{-3})	ND

Propionate[II] HMDB0000237	1.04	9.8×10^{-3} ($4.0 \times 10^{-3} - 20.3 \times 10^{-3}$)	2.7×10^{-3} ($0.0 \times 10^{-3} - 11.6 \times 10^{-3}$)	2.2×10^{-3} ($0.5 \times 10^{-3} - 13.9 \times 10^{-3}$)	0.026[ab] 0.001[ac]
Sugar region[I]	3.93	10.8×10^{-3} ($3.0 \times 10^{-3} - 17.6 \times 10^{-3}$)	3.4×10^{-3} ($1.2 \times 10^{-3} - 6.6 \times 10^{-3}$)	4.5×10^{-3} ($2.0 \times 10^{-3} - 10.3 \times 10^{-3}$)	ND
Sucrose[I] HMDB0000258	3.48	2.9×10^{-3} ($0.3 \times 10^{-3} - 5.1 \times 10^{-3}$)	3.0×10^{-3} ($1.1 \times 10^{-3} - 20.8 \times 10^{-3}$)	3.1×10^{-3} ($2.1\times 10^{-3} - 8.8 \times 10^{-3}$)	ND
Taurine[I] HMDB00251	3.42	2.2×10^{-3} ($0.2 \times 10^{-3} - 5.5 \times 10^{-3}$)	2.7×10^{-3} ($1.2 \times 10^{-3} - 39.4 \times 10^{-3}$)	4.0×10^{-3} ($1.6 \times 10^{-3} - 13.8 \times 10^{-3}$)	ND
Valerate[I] HMDB0000892	1.42	1.9×10^{-3} ($0.5 \times 10^{-3} - 4.3 \times 10^{-3}$)	1.6×10^{-3} ($0.4 \times 10^{-3} - 4.0 \times 10^{-3}$)	2.7×10^{-3} ($0.8 \times 10^{-3} - 7.8 \times 10^{-3}$)	ND

δ = chemical shift; [I]Indicates which metabolites showed statistical difference ($p \leq 0.05$) in the multivariate analysis (VIP score of the PLS-DA analysis); [II]Indicates which metabolites showed statistical difference in the univariate analysis; The statistically significant metabolites were presented above according to multivariate analysis (VIP score of the PLS-DA analysis) between the PCR+, post SARS-CoV-2 infection, and PCR- groups, their respective chemical shifts and the significance value obtained in the univariate analysis. The ANOVA test with Bonferroni correction was performed ($p < 0.05$) for the comparisons (PCR- x PCR+, PCR- x post SARS-CoV-2 infection, PCR+ x post SARS-CoV-2 infection) indicated by the superscript letters, respectively ([a, b, c]). CI = confidence interval; [a]PCR-, [b]PCR+, and [c]Post SARS-CoV-2 infection.
